# Provider Advice About Weight Loss in a Primary Care Sample of Obese and Overweight Patients

**DOI:** 10.1177/2150131917715336

**Published:** 2017-06-23

**Authors:** Chanita Hughes Halbert, Melanie Jefferson, Cathy L. Melvin, LaShanta Rice, Kemi M. Chukwuka

**Affiliations:** 1 Medical University of South Carolina, Charleston, SC, USA

**Keywords:** provider advice, weight loss and management, shared decision making

## Abstract

**Objective:** Primary care providers play an important role in obesity prevention and reduction by advising patients about weight loss strategies. This study examined receipt of provider advice to lose weight among primary care patients who were overweight and obese. **Methods:** Observational study conducted among primary care patients (n = 282) who completed a survey that measured receipt of provider advice about weight loss/management, chronic health conditions, perceived weight status, and perceptions about shared decision making about weight loss/management. **Results:** Fifty-nine percent of participants had been advised by their physician to lose weight. Participants who were obese were more likely than those who were overweight to report provider advice (odds ratio [OR] = 1.31, 95% CI = 1.25-4.34, *P* = .001). Similarly, participants who believed they were obese/overweight had a greater likelihood of reporting provider advice compared with those who did not believe they were obese/overweight (OR = 1.40, 95% CI = 2.43-6.37, *P* = .0001). Shared decision making about weight loss/management was associated with an increased likelihood of reporting provider advice (OR = 3.30, 95% CI = 2.62-4.12, *P* = .0001). **Conclusions:** Patient beliefs about their weight status and perceptions about shared decision-making are important to receiving provider advice about weight loss/management among primary care patients. **Practice Implications:** Continued efforts are needed to enhance provider advice about weight loss/management among obese/overweight patients.

## Introduction

Obesity and excess body weight are significant clinical and public health issues that disproportionately affect racial and ethnic minorities and other medically underserved populations.^1^ In addition, being obese and having excess weight is a risk factor for many chronic diseases that include diabetes, cardiovascular disease, hypertension, and some forms of cancer.^2^ Health care providers are essential facilitators to preventive care and disease management, and play an important role in helping patients to adopt and maintain healthy lifestyle behaviors that are necessary for obesity prevention and reduction. For this reason, providers should screen all patients for obesity and offer lifestyle counseling and/or refer patients to community-based programs that are designed to facilitate health behavior change.^3^ Physician discussion about a patient’s weight status has been associated with patients reporting a 5% weight loss during the past year.^4^ However, previous research has shown that only a little more than half of obese patients are advised by the health care provider to exercise and disparities exist in provider advice about obesity reduction and prevention behaviors.^5^ Furthermore, the rates of physician counseling about weight loss has declined overall from 7.8% to 6.2% of office visits and only 42% of primary care providers in a national sample performed any weight counseling with patients.^6,7^ Prior studies have also examined receipt of provider advice about weight loss among primary care patients based on their income level and practice setting. For instance, Davis et al^8^ found that patients from high-income households were most likely to report that they received advice about dietary behaviors from their providers compared with patients from low- and middle-income households. Other work has shown that prior to communication skills training, only 23% of low-income patients receiving medical care in a public health nephrology clinic recalled that their health care provider recommended that they lose weight.^8^ Similarly, only 48% of respondents in a state-based random digit dial household survey reported that their health care provider had advised them to lose weight.^9^ But, limited empirical data are available on psychological factors that are associated with patient report of provider advice about weight loss.

The purpose of this study was to examine receipt of physician advice about weight loss or management from the perspective of primary care patients who were overweight or obese. This study extends previous research that measured physician advice about weight loss in low-income and general population samples^8,9^ by examining the relationship between provider advice and psychological factors such as self-efficacy for weight loss, perceptions about weight, and beliefs about shared decision making (SDM) about weight loss/management in a racially and economically diverse sample of primary care patients. SDM is a critical component of patient-centered care^8^; components of SDM include information sharing during which patients and providers discuss symptoms and treatment options, deliberate about treatment options and discuss patient’s concerns and preferences, and make decisions that are based on the provider’s recommendation, the patient’s self-efficacy, and their ability to implement their provider’s recommendation. Previous research has shown that SDM is associated with improved health outcomes^10,11^; provider advice about weight loss/management is an important first step in patient’s weight loss and management behaviors. For this reason, SDM should be higher among patients who report that their providers advised them to lose/manage weight relative to those who do not report provider advice.

## Materials and Methods

### Study Population

We generated a convenience sample consisting of primary care patients who were either overweight or obese (n = 282) to participate in a cross-sectional study of receipt of provider advice about weight loss. Obesity and overweight status was determined using the Centers for Disease Control definition; patients who had a body mass index (BMI) between 25.0 and 29.9 kg/m^2^ were classified as being overweight and those who had a BMI ≥30 kg/m^2^ were classified as obese.^12^ To be included in the study, patients had to have at least a 3-year history of being a patient in the practice and be at least 18 years of age. Participants were recruited from primary care practices that were members in a national practice-based research network (n = 7) or part of federally qualified health care system based in the Southeastern part of the United States (n = 1). The mean number of participants enrolled from each practice was 31.3. This study was approved by the institutional review board at the Medical University of South Carolina.

### Procedures

**Figure 1.**
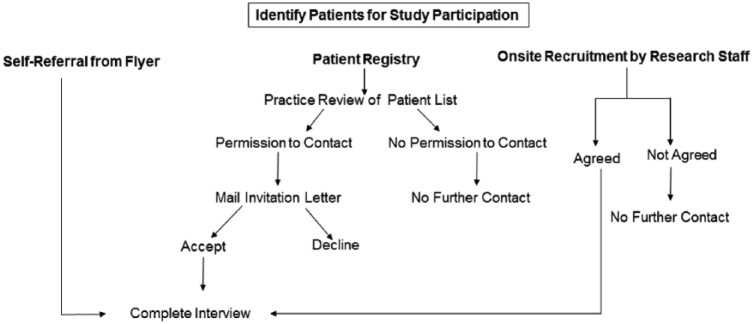
Overview of study procedures.

Patients were recruited from primary care practices using 1 of 3 mechanisms (see Figure 1). To enhance participation, more than one recruitment strategy could be used in practices. First, patients could self-refer for study participation from study flyers that were posted in the practices or given to patients when they checked in for clinic appointments. The flyer described the study as research that was being conducted to understand patient preferences for weight loss interventions in primary care and to understand barriers and facilitators to weight loss in these settings. The study flyer directed patients to contact a toll-free number if they were interested in learning more about participation. Patients who self-referred from the study flyer were screened for eligibility using a structured script and those who were eligible completed the interview by telephone after providing verbal informed consent. Patients could also self-refer for participation from study information that was posted on the practice portal. The information posted on the practice portal described the study in the same way as the flyer and also directed patients to use the toll-free number to contact the research team if they were interested in participating. Patients who self-referred from the patient portal were also screened for eligibility and those who were eligible and interested in participating provided verbal informed consent prior to completing the interview by telephone. Self-referral from the study flyer or patient portal was used by 3 (33%) practices. Patients were also identified from a practice registry maintained by the research network. Following identification, these patients were mailed an invitation letter that described the purpose of the study, the procedures involved in participation, and a self-addressed reply card that could be returned if they wanted to opt-out of being contacted for the study. Patients could also opt-out of the study by calling a toll-free study number or emailing a study research assistant. Those who did not opt-out were contacted for the interview. The interview was completed by telephone after patients provided verbal informed consent for enrollment. This method was used by 7 of the 9 practices (78%). Last, patients were approached for participation on-site by research staff during clinic hours and invited to participate in the study. This method was used at 2 (22%) practices. The purpose of the study and the procedures involved in participation were described to these patients and those who were interested in participating were administered the interview in a private area after providing written informed consent. The interview was a structured 20-minute survey that obtained sociodemographics, clinical characteristics, psychological variables, and receipt of physician advice about weight loss. The survey was administered in Spanish either in-person or by telephone to those who did not speak English. Patients were given a $25 gift card after completing the interview regardless of the method through which they were recruited.

### Measures

The measures described below were obtained by self-report during the survey. The survey was conducted from December 2015 to January 2017.

#### Sociodemographic Characteristics

Race, gender, age, marital status, education level, employment status, and income level were obtained by self-report using items from our previous research.^13^

#### Clinical Factors

Height and weight were obtained by self-report and this information was entered into the National Institutes of Health BMI calculator to determine obesity status. Participants who had a BMI that was ≥30 kg/m^2^ were categorized as being obese and those who had a BMI between 25.0 and 29.9 kg/m^2^ were categorized as being overweight. Participants were also asked if they had a personal history of diabetes or hypertension (yes or no) using items from the Behavioral Risk Factor Surveillance Survey.^14^

#### Psychological Characteristics

Participants were asked if they were ready to make weight loss or maintenance efforts and how confident they were in their ability to lose or manage weight using 2 Likert-style items. These items were adapted from our previous research on integrated risk factor education.^15^ Specifically, participants were asked how ready they were to start trying to lose weight or keeping their weight the same (1 = not at all/have not thought about it, 2 = a little ready, 3 = somewhat ready, 4 = very ready, 5 = already trying to lose weight/keep weight the same) to measure readiness for weight loss. Responses to this item were re-coded into a dichotomous variable that reflected ready (very ready/already trying to lose or keep weight the same) or not ready (not at all/a little ready/somewhat ready). In addition, participants were asked how confident they were that they can lose weight or keep their weight the same (1 = not at all confident, 2 = a little confident, 3 = somewhat confident, 4 = very confident, 5 = completely confident). Responses to this item were also recoded into a dichotomous confidence (very/completely) or not confident (not at all/a little/somewhat) variable.

#### Shared Decision Making

The SDM-Q-9 was used to measure SDM about weight loss/management.^16^ Minor adaptations were made to the introduction of this instrument to increase its relevance to weight loss/management. Specifically, the introduction to the SDM scale read as “Thinking about the last time you talked with your health care provider about your weight, and things that you could do to lose weight or keep your weight the same, how much did your provider . . .” Following this introduction, the original SDM items (eg, made clear that a decision needs to be made, wanted to know exactly how I want to be involved in making the decision, and told me that there are different treatment options) were administered. One item (my doctor and I thoroughly weighed the different treatment options was omitted because of an administrative error; nevertheless, this scale had good internal consistency (Cronbach’s α = .94). Items were summed to calculate a total score for SDM; higher scores indicated greater SDM.

#### Physician Advice About Weight Loss

Participants were asked to indicate if they had ever been advised by a doctor or other health care professional to lose weight or reduce their weight (yes, no, or do not know). Responses of “do not know” were categorized as “no” in the statistical analysis. Similar items have been used in previous population-based surveys to measure receipt of physician advice about weight loss.^17^

### Statistical Analysis

First, descriptive statistics were generated to characterize the study sample in terms of sociodemographic characteristics, clinical factors, psychological characteristics, and receipt of physician advice about weight loss. Next, we conducted a binomial test to determine if the proportion of participants who were reported provider advice differed significantly from 50%. With a sample of 282 participants, we had 86% power to determine if the observed proportion of patients who were advised to lose weight differed significantly from the null hypothesis in which 50% participants were advised to lose weight with type I error of 0.05. We then conducted bivariate analyses consisting of chi-square tests of association and *t* tests to examine the relationship receipt of physician advice and sociodemographic variables, clinical factors, psychological characteristics, and SDM. Last, multivariate logistic regression analysis was conducted using generalized estimating equations to identify factors having significant independent associations with receiving physician advice to lose weight, while adjusting for correlations among patients from the same practice. Variables that had a bivariate association of *P* < .10 with physician advice were included in the multivariate logistic regression model. Data were analyzed using SAS Version 9.4.

## Results

Table 1 shows the characteristics of the study sample (n = 282). Sixty-five percent of participants were female, 55% were married, 57% had some college education or were college graduates, 54% were employed, and 63% had an annual household income that was greater than $20,000. Sixty percent of participants were from racial and ethnic minority groups. The mean (±SD) age of participants was 53.1 (±14.4) years.

**Table 1.** Sample Characteristics and Bivariate Analysis of Physician Advice About Weight Loss (n = 282).^a^

Variable	Level	n (%)	% Physician Advice	χ^2^	*P*
Race	Minority	168 (60)	58	0.10	.76
	Nonminority	113 (40)	60		
Gender	Male	98 (35)	46	10.40	.001
	Female	184 (65)	66		
Marital status	Married	153 (55)	56	1.15	.28
	Not married	123 (45)	63		
Education level	≥Some college	158 (57)	64	3.39	.06
	≤High school	119 (43)	53		
Employment status	Employed	148 (54)	54	3.61	.06
	Not employed	127 (46)	65		
Income level ($)	>20 000	146 (63)	59	0.76	.38
	<20 000	85 (37)	65		
Diabetes	Yes	68 (24)	72	6.59	.01
	No	213 (76)	54		
Hypertension	Yes	164 (58)	63	2.71	.10
	No	117 (42)	53		
Obesity status	Obese	181 (64)	69	21.69	.0001
	Overweight	101 (36)	40		
Perceived obesity	Yes	170 (60)	76	51.19	.0001
	No	112 (40)	33		
Ready for weight loss	Ready	206 (73)	64	8.58	.003
	Not ready	76 (27)	45		
Confidence for weight loss	Confident	169 (60)	59	0.02	.90
	Not confident	113 (40)	58		

a n may not equal 282 because of missing data.

Overall, 59% of participants had been advised by their physician to lose weight and 41% had not received this advice. The percentage of participants who reported provider advice was statistically different from 50% using the binomial test (*P* = .0035). Table 1 shows the results of the bivariate analysis of provider advice about weight loss. Women were significantly more likely than men to report that their provider had advised them to lose weight. Both actual and perceived obesity were associated significantly with receiving provider advice about weight loss. Participants who were obese were more likely to report that their provider had advised them to lose weight compared with those who were overweight. Similarly, participants who believed they were obese were more likely to report weight loss advice from their provider compared those who did not believe they were obese. Diabetic patients were also significantly more likely than nondiabetic patients to report that their provider had advised them to lose weight. Last, participants who reported greater readiness to lose or maintain their weight were more likely to report provider advice about weight loss compared to those with less readiness. While employed patients, those who had at least some college education, and were hypertensive were more likely to report provider advice compared to those who were unemployed, had less education, and were not hypertensive, these associations were not statistically significant (see Table 1). There were no racial/ethnic differences in receipt of provider advice to lose weight. However, participants who reported provider advice had significantly greater SDM (Mean [SD] = 21.8 [7.0]) about weight loss compared with those who did not receive provider advice (Mean [SD] = 13.7 [7.0]) (*t* = −9.49, *P* = .0001).

The results of the multivariate logistic regression analysis that was generated using generalized estimating equations are provided in Table 2. Obesity status, perceived obesity, and SDM about weight loss/management had significant independent associations with receiving physician advice about weight loss. Participants who were obese were more likely than those who were not obese to report receiving provider advice (odds ratio [OR] = 1.31, 95% CI = 1.25-4.34, *P* = .001). Similarly, participants who believed they were obese/overweight had a significantly greater likelihood of reporting provider advice compared with those who did not believe they were obese/overweight (OR = 1.40, 95% CI = 2.43-6.37, *P* = .0001). The likelihood of reporting physician advice was also increased with greater SDM about weight loss/management (OR = 3.30, 95% CI = 2.62-4.12, *P* = .0001). We created an interaction term using hypertension and diabetes status to determine if patients who had both these conditions were more or less likely to report provider advice about weight loss; this interaction was not statistically significant (*P* = .32) (data not shown in table).

**Table 2.** Multivariate Logistic Regression Model of Physician Advice About Weight Loss.

Variable	Level	Odds Ratio	95% Confidence Interval	*P*
Gender	Male Female	1.10	0.67-2.60	.41
Education level	≥Some college ≤High school	1.55	0.97-6.37	.06
Employment status	Employed Not employed	0.82	0.25-1.33	.13
Diabetes	Yes No	1.35	0.87-4.69	.10
Hypertension	Yes No	0.96	0.50-1.50	.60
Obesity status	Obese Overweight	1.31	1.25-4.34	.001
Perceived obesity	Yes No	1.40	2.43-6.37	.0001
Ready for weight loss	Ready Not ready	1.03	0.66-1.88	.69
Shared decision making	^a^	3.30	2.62-4.12	.0001

a Odds ratio for continuous variable represents 1 SD unit change.

## Discussion

The purpose of this study was to examine receipt of physician advice to lose or manage weight from the perspective of primary care patients who were overweight or obese. Overall, 59% of participants reported that providers had advised them to lose/manage their weight and 41% were not advised to lose/manage their weight. The rates of physician advice about weight loss that were reported in the present study are higher than those reported by participants in prior studies. For instance, 42% of participants in a population-based study reported that their health care provider had advised them to lose weight.^17^ Similarly, 39% of obese patients reported that their providers had advised them to lose weight.^18^ Brietkopf et al^19^ found similar rates of physician advice among women who had low socioeconomic resources. While our rates of physician advice about weight loss are higher than those previously reported, our findings demonstrate that there is room for improvement with respect to physician advice about weight loss. All the participants in this study were either overweight or obese, and guidelines from the US Preventive Services Task Force recommend that health care providers screen all patients for obesity and offer weight loss interventions or refer obese patients to weight loss programs.^3^ Based on this recommendation, it would be expected that rates of physician advice about weight loss/management would be higher in our sample because participants were obese or overweight. Providers may not be advising patients to lose/manage weight because of time constraints during the medical visit. Twenty-four percent of patients had diabetes and 58% had hypertension; greater emphasis may be placed on managing these and other chronic conditions. Another possible explanation for the modest rates of provider advice to lose/manage weight is that intensive, multicomponent interventions are recommended for obesity reduction and prevention.^3^ Providers may not see the value in offering advice about weight loss/management outside of these types of programs. Previous research has shown that providers may not feel prepared to address weight loss/management among patients and also lack knowledge about weight loss targets for obese and overweight patients^20,21^; this may explain why patients are not being advised to lose or manage their weight.

We also found that the participant’s obesity status and perception about their weight had significant independent associations with reporting provider advice about weight loss/management. Participants who were obese were about more likely than those who were overweight to report provider advice about weight loss/management. Obesity is now recognized as a chronic condition in addition to being a risk factor for diabetes, hypertension, cardiovascular disease, and some forms of cancer. Thus, even though rates of provider advice reported by participants in our study may be modest, our findings suggest that providers are targeting their efforts for advising patients about weight loss/management to the patients who are at increased risk for adverse health outcomes because of their weight. This approach is consistent with the US Preventive Services Task Force recommendations, but raises the question of if providers should wait until patients cross the body mass index threshold for obesity before offering advice about weight loss/management. Providers may recognize the potential for overweight patients to become obese; this may explain why 40% of overweight participants in our study reported that they had been advised by their provider to lose or maintain their weight. This practice may reinforce patient beliefs about their weight. Sixty-nine percent of participants in this study believed they were overweight/obese and were more likely than those who did not believe they were overweight/obese to report that their providers had advised them to lose weight. These patients may have more realistic beliefs about their weight status and be more receptive to provider messages about weight loss and management.

We also found that patients who perceived greater SDM about weight loss/management with their providers were about 3 times more likely to report physician advice. SDM is a critical component of medical care that reflects the extent to which patients believe that their provider gave sufficient information, deliberated and negotiated options with their provider, and ultimately, reached a decision together with their provider.^22^ In essence, SDM is an indicator of the quality of provider communication about a clinical issue. Participants who reported SDM about weight loss/management indicated that providers were likely to make clear that a decision needs to be made, discuss different treatment options, explain the advantages and disadvantages of options, help them to understand this information, and reach an agreement with them on how to proceed. Previous research has shown that better quality communication about weight loss is associated with improved patient recall of weight loss recommendations and improved confidence to lose weight.^8^ Additional research is needed to determine if SDM about weight loss/management is associated with patients making efforts to lose/manage their weight.

In considering the results of this study, several limitations should be considered. First, the cross-sectional nature of the study does not enable us to determine causality with respect to receipt of provider advice and SDM, psychological variables, clinical factors, and sociodemographic characteristics. In addition, we measured receipt of provider advice about weight loss/management by self-report from the perspective of patients. This approach may be subject to patient recall bias. We did not ask patients about the specific advice they were given by providers nor did we observe delivery of provider advice to patients. In addition, participants were not asked about the number to times that weight loss advice had been given by their health care provider. These are important issues that should be examined in future studies. However, the item we used to measure provider advice about weight loss/management has been used in several prior studies and has acceptable face validity. Nonetheless, additional research should be conducted to determine the concordance between patient’s report of physician advice about weight loss/management and documentation of the specific content of these discussions in the patient’s medical record.

## Conclusions

Despite the potential limitations described above, our study sheds new light on variables that are important to patients being advised by providers to lose/manage their weight. The results of this study demonstrate that many overweight/obese patients may not be receiving advice to lose/manage their weight by their health care provider. While providers should advise overweight/obese patients about weight loss and management, patient beliefs about their weight status and perceptions about shared decision-making are important to reporting provider advice about weight loss/management was received among primary care patients. Patient beliefs as well as provider behaviors should be addressed as part of efforts to improve the management of obesity/overweight in primary care.

## Practice Implications

Previous research has shown that provider advice to lose weight is associated with greater confidence for weight loss among patients; education about obesity prevention and control and communication skills training improves the rates of provider counseling about weight loss.^8^ Other work has shown that overweight and obese patients who are informed that they are overweight by providers are likely to make weight loss efforts.^4^ Our findings demonstrate that continued education and communication skills training are needed to increase rates of provider advice about weight loss/management among primary care patients who are overweight or obese. The results of the present study suggest that interventions that target provider knowledge and communication skills about obesity prevention may need to address constructs from theoretical models of patient behavior change. Forty percent of patients did not consider themselves to be overweight or obese, 27% were not ready to make weight loss/management efforts, and 40% had low confidence in their ability to lose/manage their weight. Perceived risk is an important component of the health belief model^23^; patient’s perceptions about their obesity status was associated significantly with patient report of provider advice about weight loss in our multivariate logistic regression model. Patient-centered counseling about obesity reduction and/or prevention that includes provider advice about weight loss/management may need to begin by increasing patient’s awareness about his or her obesity status and the disease risks of associated with being overweight or obese.^24^ Although readiness for weight loss/management did not have a statistically significant association with reporting provider advice about weight loss in the multivariate logistic regression model, recommendations for patient-centered counseling for behavior modification emphasize assessing the patient’s readiness to change.^24,25^ Assessing the patient’s readiness for change is consistent with the transtheoretical stages of change model^26^; understanding patient readiness would enable providers to offer advice about weight loss that is tailored to the issues that patients are facing at different stages of readiness. Strategies are also needed to document the provision of this advice in the patient’s medical record. But, addressing provider behavior alone is not likely to be sufficient. As noted earlier, readiness for weight loss and perceived obesity status were associated with receipt of provider advice about weight loss/management in the bivariate analysis and perceived obesity/overweight had a significant independent association with provider advice in the multivariate logistic regression analysis. Interventions may be needed to improve patient’s perceptions about their weight in order to increase the saliency of provider advice about weight loss.
